# The experiences of people living with HIV/AIDS and of their direct informal caregivers in a resource-poor setting

**DOI:** 10.1186/1758-2652-13-20

**Published:** 2010-06-11

**Authors:** Basanti Majumdar, Nomathemba Mazaleni

**Affiliations:** 1 Master University, School of Nursing and Department of Family Medicine, Hamilton, Ontario, Canada; 2 Management Sciences for Health, Center for Health Systems and Services, East London, Eastern Cape, South Africa

## Abstract

**Background:**

HIV/AIDS is a critical concern in South Africa, where extreme poverty and gender issues are major determinants of health. A comprehensive home-based care programme is needed to lessen the burden placed on the caregivers of those suffering from HIV/AIDS. The purpose of this study was to explore and describe the challenges faced by people who are living with HIV/AIDS and by their caregivers in resource-poor, remote South African villages.

**Methods:**

In-depth interviews were conducted with nine persons living with HIV/AIDS and their nine direct informal caregivers. Interviews explored the themes of physical, emotional and social wellbeing. Two focus groups, one involving community leaders and one with local health care providers, were conducted to build on themes emerging from the in-depth interviews. A thematic analysis of the transcripts was performed.

**Results:**

This study sheds light on the needs of persons living with HIV/AIDS and the needs of their direct informal caregivers in a rural area of the Eastern Cape province of South Africa. These needs include: physical/medical, social, material, financial, physiological/emotional, gender issues, and instrumental.

**Conclusions:**

In developing home-based care programmes, it is vital to consider the perceived needs of persons living with HIV/AIDS and their direct informal caregivers. The results from this study serve as a basis for the development of a home-based care programme in one resource-poor setting of South Africa and could provide a model for such programmes in similar areas.

## Background

The latest *AIDS Epidemic Update*, published by the Joint United Nations Programme on HIV/AIDS (UNAIDS) and the World Health Organization (WHO), estimates that 33.2 million people were living with HIV in 2007 [1]. With an estimated 5.5 million people infected [2], South Africa is burdened with the largest HIV-infected population in the world [1].

Although South Africa is considered to be a middle-income country, it has one of the highest levels of income inequality in the world. While the wealthiest 10% of the population account for 51% of the country's income, the poorest 10% account for only 0.2% [3]. Despite post-apartheid policies that have increased health spending in poor districts, rural and African residents bear the largest burden of illness [3,4]. Conditions created by apartheid, such as migrant labour and underdeveloped health services for African people, have resulted in an environment that allows for the efficient transmission of HIV [3,5].

The rapid spread of HIV throughout SA has created a burden on the already underdeveloped public health care delivery system [4]. Hospitals and tertiary care facilities are becoming increasingly unable to care for their HIV/AIDS patients [6]. Although post-apartheid policies have aimed to redistribute spending to primary health care, this section of the health system remains inadequately funded and resourced [3-5,7]. Consequently, much of the burden of caring for those infected with HIV/AIDS falls onto households and communities [8-10].

In its Operational Plan for Comprehensive HIV and AIDS Care, Management and Treatment for South Africa the South African government (2003) stated that one of its major goals was to provide adequate community-based treatment, care and support services for people living with HIV/AIDS (PLHIV) before the end of the decade [11]. Community-based home care (CBHC) programmes in the country have since been bolstered by increased government grants and training for community health workers [12]. CBHC services are an integral and affordable part of a national strategy to address the multi-dimensional needs of those affected by HIV/AIDS [2,8,13].

Support services for PLHIV and their direct informal caregivers (DICGs) must be developed based on research examining: the current state of accessible health care in their communities; the health conditions of PLHIV; and mitigating social factors.

The purpose of this paper is to describe the major findings of a qualitative descriptive study conducted in 2002. The study identified the needs of PLHIV and their DICGs through an examination of the day-to-day experiences of PLHIV in rural Eastern Cape, South Africa. The needs of PLHIV and their DICGs have formed the foundation for the development of a model of community-based home care that could be applied in order to alleviate some of these needs. The scope of this paper is to present the identified needs of the PLHIV and their DICGs.

## Methods

### Purpose

The purpose of the current study was to explore and describe the challenges faced by PLHIV and their DICGs in two remote South African villages, and their ability to cope with the illness given the scarce social and economic resources within their communities.

### Ethics

Research Ethics Boards in Canada and South Africa granted approval for this project. Nurses from the local health centres held community meetings to inform community members of the study. Additionally, the study received approval from the community chief and elders. All participants gave their written informed consent prior to participating in the study. Consent forms were written in the local language of isiXhosa.

### Timeline

Data collection took place in 2002 during a three-week period.

### Study site

The project was conducted in two isolated rural townships of the Eastern Cape, an impoverished province with a high unemployment rate. In 2000, the Eastern Cape was home to 6.82 million people, with 85% of the population being African and 65% of the population living in rural areas [14]. The poverty rate in the Eastern Cape (68.7%) is the highest in the country. A wide disparity exists among the population: there are very low levels of poverty among white people, but a 73.8% rate among African people [14]. Poverty is also a rural phenomenon in this province, with the rural poverty rate estimated at 82.2%, compared to 42.1% in urban areas [14].

A high prevalence of HIV is found in the Eastern Cape. By mid-2006, 19.2% of adults aged 20-64 (17.2% men vs. 20.9% women) in the province were living with HIV [15].

The two rural townships selected for this study represent isolated communities facing problems of HIV/AIDS and poverty, and thus could serve as models for similar areas. The two sites had populations of 18,519 (Township A) and 6,845 (Township B). Township A was equipped with a primary health care centre with full-time nursing staff. Township B was equipped with a community care centre, staffed by community members and visited by a nurse three days per week.

These government-funded clinics are mandated to meet the immediate health care needs of the area. Individuals who require the care of a physician must travel to the closest government hospital. While visits to the local clinics were free, at the time of this study, a visit to the local hospital cost eight rand (R), about US$1.

### Sample selection

In each rural township, community workers from the local health centre recruited PLHIV and their respective DICGs to participate in in-depth, individual interviews. PLHIV selected for participation met the criteria that: (a) they had been diagnosed with HIV; and (b) they could identify one person who provided them with informal care. Each participating PLHIV identified a person who provided him or her with informal care for inclusion in the study.

### Data collection

Semi-structured interviews were conducted concurrently with each PLHIV and his or her DICG by two graduate students working with the research team. Interviews were conducted using an interview guide containing both close-ended demographic questions and open-ended questions addressing the themes of physical, mental and emotional health. To limit intrusion into private households and protect confidentiality, participants were given the choice of being interviewed in their home or at the local health centre.

Four community workers from the local health centres translated consent forms and interview guides from English to isiXhosa, and acted as translators during interviews. These four volunteers were fluent in both English and isiXhosa. At the end of the interview, participants received a food package as an appreciation for their time. Participants were not told ahead of time that they would receive this food package.

### Focus group interviews

Two focus group interviews were conducted to build on themes emerging from the in-depth interviews. Participants of the first focus group had a background in health care and experience in caring for patients with HIV/AIDS. Participants of the second focus group were identified as leaders in their community. Focus group interviews were conducted by two graduate students working with the research team, using interview guides written in English. The health care provider focus group was conducted in English and lasted for two hours. The community leader focus group was conducted in isiXhosa, with community workers from the local health centre acting as translators, and lasted for four hours.

### Data analysis

All interviews and focus groups were tape-recorded with the permission of the participants. Tapes were transcribed verbatim, and those conducted in isiXhosa were translated to English. Local research team members carried out translation and validation of the transcripts with the assistance of the translators. Four members of the research team, two from Canada and two from South Africa, independently coded and categorized the data. A very close level of agreement was observed between researchers. Final themes were decided by consensus. Local project team members then validated the themes with participants and community stakeholders.

## Results

### Participant characteristics

### Persons living with HIV/AIDS

Seven female and two male PLHIV between the ages of 28 and 42 years participated in the study. The proportion of female participants outweighed male participants due to the fact that many males stated that they were not comfortable being interviewed during the recruitment process.

Eight participants were single, divorced or widowed, and one was married. All participants, except one, reported that they were diagnosed with HIV within the past two years. The other participant had been diagnosed with HIV for six years. Four reported that they contracted HIV through unprotected sexual intercourse and five did not know how they became infected. All participants were unemployed and dependent on government disability grants (referred to by participants in this study as "pensions") or support from family members. Five PLHIV had recently received or were currently receiving treatment for tuberculosis.

### Direct informal caregivers

All nine participating caregivers were immediate female family members of the PLHIV and ranged in age from 38 to 74 years. Most were married and had children and a family to care for, apart from the PLHIV and, in some cases, the children of the PLHIV. All DICGs relied on government financial assistance (disability grant, old-age pension, or care-dependency grants) via a monthly payment of R620 ($78). The caregivers represented in this study reflect others from published literature, which have reported that DIGCs in South Africa tend to be female family members who are living below the poverty line [16-18].

### Focus group participants

The eight participants of the first focus group had a background in health care and experience in caring for PLHIV. The backgrounds of the participants included a doctor, a social worker, a health promoter from the Provincial Department of Health, three nurses from the local health centres, the matron and supervisor of the health centres, and a teacher and coordinator of the primary school education programme which provides support to children affected by HIV/AIDS.

The 18 participants of the second focus group were identified as community leaders. Participants included 13 respected elders, one religious official, one political councillor, and three volunteer HIV/AIDS workers. The community leader focus group was larger than the traditional size of six to 12 members in order to accommodate the numbers of community leaders who expressed interest in participating.

#### Themes from the data

Themes that emerged through in-depth interviews with PLHIV and DICGs, as well as the two focus groups, can be broadly categorized as: (1) needs of the PLHIV and their DICGs; and (2) necessary components of a community-based home care programme. The focus of this paper is to present the needs of the PLHIV and their DICGs. These needs include: physical/medical, social, material, financial, physiological/emotional, gender issues, and instrumental.

#### Physical/medical issues

All PLHIV reported deterioration in physical health due to their illness. Most suffered from lack of appetite, tiredness and pain. Participants reported that they visited the local health centre if they felt ill, and received medication to treat their symptoms. None of the PLHIV were undergoing antiretroviral treatment at the time of the interviews. Diarrhoea, fatigue, pain, rashes, and headaches were common symptoms and deteriorating health made it difficult for some participants to maintain their pre-diagnosis activity level:

I used to do everything in my house myself. Now, I feel pain in my feet and I get tired. (PLHIV)

I forced [myself] to make [things] but sometimes, the body becomes very painful. (PLHIV)

Focus group participants also spoke about their personal experiences caring for family members infected with HIV/AIDS and the associated medical symptoms:

But the problem is that when she eats, she has a loose stool and that makes her weak. She has no strength. She has lost weight and sometimes she even vomits. (community leader focus group participant)

In addition to the deteriorating physical health that PLHIV experience, DICGs also reported stress-related physical symptoms, including headaches, lack of sleep, body pain and abnormal blood pressure. One DICG explained the burden put on her to continue providing care despite the toll on her personal health:

I am tired all the time and my body is full of pains. Even if I am tired, I say that I must continue the work because there is nobody else who is taking care of them. (DICG)

PLHIV and their DICGs expressed an obvious need for physical and medical assistance to cope with symptoms of HIV/AIDS, but interviewees could name few community resources available to assist them. Although participants named the local health centre as their primary source of medical assistance, participants also described a shortage of staff within the centre and an occasional shortage of medications. According to one participant in a community leader focus group, "The clinic is short of medicine. Sometimes you get help, sometimes you don't."

In addition to sporadic support from the clinics, participants stated that PLHIV living in their communities were not receiving assistance at home from nurses or other trained health care workers.

#### Psychological/emotional Issues

Many PLHIV reported sadness and worry as a result of their deteriorating health, the grave prognosis, and the fate of their children:

Sometimes I feel sad...My health gives me some problems. I am always thinking of my status. (PLHIV)

Now the days are [worse] than before. I always think of the disease...(PLHIV)

However, while some respondents exhibited despair, others retained some hope:

I am thinking of my life ahead in the future because I know it will not be the same as it used to be. I have good feelings about the future even if I don't know if I will be there. (PLHIV)

PLHIV coped with the psychological burden that HIV/AIDS created by learning to accept their status, talking to family, or drawing support from religion and spirituality. As one interviewee stated, "Most of the time, I can't sleep. But after I open up, I feel much better." Another reported, "Happiness comes in terms of love. I am loved by family members."

During the interviews, DICGs professed love and support for the PLHIV, and felt that their care was better than no care at all. As a DICG said, "I am doing everything because I have to do it. There is nowhere to run away from the responsibility. I have accepted it as it comes."

However, caring for the PLHIV results in barriers to socializing and restricts support networks available to the DICG. This isolation results in a large emotional burden for the DICGs:

Due to my emotions I am no longer socializing...Because I just sacrificed for the situation and taking care of my family, my husband and my children. Sometimes I am depressed but have to adjust myself so that I can go on helping them because I love them so much. (DICG)

Other caregivers explained:

I don't want to lose hope because I could leave her behind and die before she does. There is time. (DICG)

There [are] times when I feel...I do not know where to start and where to end. At this point I turn to God to help us. (DICG)

The psychological and emotional impact of HIV/AIDS on the PLHIV and his or her DICG can be substantial and must be addressed. No participants identified the clinic as a source of emotional support, and researchers were told that the clinic was short staffed.

PLHIV and DICG identified religion as being very important. However, the religious organizations within the community did not appear to reach out to their impoverished congregations. Participants from the community leader focus group identified that churches are beginning to address the psychological and emotional needs of PLHIV. However, PLHIV who mentioned "church" as a source of support stated they received only spiritual support, and most had not disclosed their status to the priest or congregation.

#### Social issues

Stigma and isolation were important issues for PLHIV. Living in a small rural community, participants found it difficult to hide their HIV status, particularly as their disease progressed:

People with HIV/AIDS are neglected. You are judged. They are insulting the person. They hate her. They don't want to come near her. (PLHIV)

One PLHIV became very emotional and recalled:

They are insulting me. They say I've got the sore mouth because I've got AIDS...The community needs to be taught how to handle the people with HIV, because they are always laughing at them and insulting them. When they are talking together and they see me and they are talking about me, this is why I don't tell anybody. (PLHIV)

As a result of the stigma associated with HIV/AIDS in the community, support from friends was minimal; several of the PLHIV stated that they had no friends or that their friends were unsupportive. PLHIV and DICGs pointed to the association between HIV and sexual intimacy as the possible root of stigma. As one PLHIV said, "We are blamed and taken as prostitutes." Focus group participants also attributed the stigma attached to HIV/AIDS to its association with sex and sexuality:

To our people, to our neighbours, it's a disgrace if your child is HIV positive, as if you are the cause of it. And sometimes you don't know from which direction it came...(community leader focus group)

They say it's because girls don't behave. Girls have a lot of boys, boys have a lot of girlfriends. And it goes on like that. (community leader focus group participant)

Others in the focus group commented on the cultural customs that restrict sexual behaviour to marital relationships, and that those engaging in sexual relations without being married are looked down upon. Furthermore, those who are married and diagnosed as HIV positive are perceived as immoral:

Our culture does not allow for relations before marriage, so when you have AIDS, you feel that you have done something wrong, unacceptable behaviour. Even for a married couple, because it means that one has not been faithful to the partner. (health care provider focus group participant)

Focus group participants also discussed the implications of this stigma, including fear of disclosure, the isolation of PLHIV by the community, and silence around HIV/AIDS:

When they hear that someone has HIV, [people] say, "I don't want to know this person. I don't want to go near this person." And they shut the doors of the community to this person. There is a lot of discrimination toward HIV-positive people. (community leader focus group participant)

Fear of stigma and disclosure was heightened among participants who discussed problems of maintaining privacy in a small, isolated, rural setting. As the participant's HIV/AIDS symptoms progressed, hiding his or her HIV status from the community became increasingly difficult.

In most cases, caregivers displayed complete devotion to the PLHIV. Though the fear of contracting HIV did cross their minds, it did not hamper their willingness to provide care:

We have welcomed our brother because we love him. There's no other way...We decided just to accept him and love him the way we loved him before. Sometimes, there's a sadness and [we are] downhearted because it was not easy for us to accept that but, at last, we accepted...(DICG)

Interview and focus group participants believed that community members' fear of infection promotes stigma. As one PLHIV said, "People don't care about us. They don't like us because they think they are going to be infected." Fear is perpetuated by a lack of education and information about HIV/AIDS in the community. Participants believed that further community education could succeed in removing the stigma attached to HIV/AIDS and increase community acceptance of those infected and affected by this illness:

We must have people from the community come together and talk and discuss and share views on how to deal with this. We must have traditional healers, doctors, and others. We have to accept people and get rid of stigma. This starts at home. We have to accept family members with HIV/AIDS. (DICG)

#### Financial

Participants attributed the extreme poverty affecting the community to the high level of unemployment, as well as other economic problems plaguing South Africa. "There's no work. Everybody has very little to live on...there's no money, and the rand is going up and down," a participant in the community leader focus group said. For those living in the area, poverty is exacerbated by drought, which is afflicting the village and rendering agricultural or farming activity nearly impossible. According to a community leader focus group participant, "Now there's a drought, we have nothing in our gardens."

All PLHIV participating in this study were unemployed and living in extreme poverty. As one PLHIV stated, "The money is too little and the family is too big." In one case, 14 people were dependent on one pension and the sporadic income of one working adult. A PLHIV stated, "...it doesn't cover to support the children, even no[t] enough food to be covered." Another PLHIV remarked, "People are very poor. I would like to have some cattle and something to plant."

A government grant of R620 was provided to PLHIV on a monthly basis. However, the processing time for granting a pension is sometimes unacceptably long and the amount of pension granted far too low to cover even basic expenses. In one case:

My daughter who died - they took seven months to get a grant but she had died by the time the grant came. I asked her brother to dig into this issue because I am debt, no money to feed the children. And they gave me only R1000. Then I applied and got the R100 and R200 per child. I can't even pay her rent with this. (community leader focus group participant)

According to participants, poverty leads to poor care and support of PLHIV as they are unable to access the treatment and nutrition necessary to maintain their health. On a broader scale, poverty affecting the area leads to a corrosion of the sense of community that once existed within the village. Community members do not have the means to lend support to neighbours who are ill. "It's very difficult to help someone when you have nothing," a community leader focus group participant said.

#### Material

Due to the financial drain and limited physical mobility created by HIV/AIDS, access to basic material needs (food, medications, hygiene products) can be a significant challenge for PLHIV and their DICGs. This lack of access is particularly problematic in areas with high poverty rates:

When we give them the education, we say, "You ought to eat a well-balanced diet." And they ask us: "Where are we going to get that well-balanced diet? With which money we can buy that food?" (health care provider focus group participant)

With poverty levels widespread throughout the community, participants were quick to point out deteriorating conditions. In addition to accessing food and medications, difficulties included affording costs associated with school, lack of child care, lack of affordable housing, and inaccessible transportation. Participants recognized that government assistance to the community in funding a CHBC system is necessary in order to provide medical support:

There are no funds for home-based care. To buy the equipment, condoms, gloves, dressing, toiletries, sheets, some incentives for the volunteers. Training people can improve home-based care and donation of equipment, linen, cotton wools, toiletries, and sheets. (health care provider focus group participant)

#### Gender issues

Community responses to HIV/AIDS reveal gender imbalances within society. One community leader focus group participant noted that people in the community believe that HIV/AIDS is spreading because "girls don't behave". Double standards also exist in which women are expected to engage in sexual relations only within marriage, but custom allows men to have multiple partners. As one health care provider focus group participant stated, "[As for the men] now they don't take so many wives; but they still have girlfriends outside the marriage."

#### Instrumental

Due to the physical limitations placed on the PLHIV by the illness, performing the tasks of day-to-day living can present a challenge. Informal caregivers provide assistance for the PLHIV in activities of daily life, including maintaining hygiene, eating and dressing. The level of care required by the PLHIV can be overwhelming:

When it comes to the brother lying on the bed, sometimes, we need to be relieved. Someone can come and help us [do the washing]. We need to have enough funds so that we can take care of the brother, provide a good diet or drugs, take care of the children, someone can come to the house and help with the washing or bathing the brother. We are receiving no help from the community. (DICG)

## Discussion

McDonnell, Brennan, Burnham and Tarantola (1994) state that in developing home-based care programmes, it is vital to consider the perceived needs of PLHIV and DICGs, and their families, even if they are difficult to meet [19]. The results of this descriptive study provide valuable insight into the needs of the PLHIV and his or her DICG living in resource-poor settings. These needs include: physical/medical, social, material, financial, physiological/emotional, gender issues, and instrumental.

Due to the debilitating nature of HIV/AIDS, the physical and medical needs of PLHIV are numerous and can be overwhelming [20]. PLHIV cope with a variety of physical problems, depending on the stage of their illness. Treatment for pain and other disease-related symptoms may require frequent travel to distant hospitals or clinics and, with the continuous deterioration of health, PLHIV require the support of trained caregivers [21]. In addition to the medical needs of PLHIV, DICGs in this study discussed physical and medical needs of their own, arising from the stress and burden of caring for PLHIV.

In the current study, the formalized medical care available in the rural communities is under-resourced and under-staffed. PLHIV and their DICGs must therefore be provided with counselling, information and support on basic medical care and infection control. Home-based medical assistance and palliative care from trained health care workers in the community has the potential to increase quality of medical care for PLHIV, as well as decrease burden and consequent health issues for DIGGs [8,13,16,22]. To achieve this, increased communication and coordination is needed between the testing centre, the health care facility, the hospice and the community in order to facilitate service utilization by the PLHIV and his or her DICG [23-27].

As many as 60% of HIV/AIDS patients suffer from major depression, often characterized by a loss of satisfaction, overwhelming sadness, feelings of guilt, and self-loathing [28]. In the current study, PLHIV who reported a high degree of integration in the community tended to be more positive and hopeful about assistance available to them and more satisfied with the assistance they received. Conversely, those PLHIV who were withdrawn received little or no assistance. This suggests that community participation increased the PLHIV's awareness of and access to community resources, and assisted in creating supportive networks.

PLHIV commonly experienced isolation, lack of acceptance, and fear of disclosure. It is well documented in the literature that stigma and discrimination are widespread and can be significant barriers to access to adequate health care, as well as psychological and social support for PLHIV and their DICGs [13,22,29,30]. This perception, perpetuated by media and public opinion, creates additional stress for infected people and makes them less likely to disclose their status and receive the support of their communities [29].

Community awareness campaigns to reduce stigma are necessary in order to promote acceptance of those affected by HIV/AIDS in the community [22,31,32]. Activities aimed at reducing stigma and the resulting isolation must also include the facilitation of access to health and social services by PLHIV and their caregivers [23-27,33]. Social and spiritual supports are important coping mechanisms for PLHIV as they promote expression of emotional and psychological distress and ease the burden of living with HIV/AIDS [22,34,35].

A system of informal and formal counselling and support must be accessible to PLHIV and their DICGs through the community, as well as through clinic- and home-based services, in order to assist them in coping with the illness. Counselling must also be provided once the PLHIV has passed away in order to support the family through the process of bereavement [22-27].

Poverty is a major contributing factor in the spread of HIV/AIDS [36,37]. South Africa, currently facing the highest HIV prevalence in the world, has one of the world's largest income disparities [15]. As demonstrated in this study, poverty poses a significant barrier to health care, since it reduces a family's ability to provide basic necessities. Problems of poverty are exacerbated when caregivers and income generators take ill and are unable to provide for other family members, particularly when government financial support is lacking [16].

A system of home-based care, which is accessible free of charge, is imperative. Social welfare must be provided for PLHIV and their DICGs in order to lessen the financial toll of this illness. Furthermore, income-generating activities must be developed in order to allow PLHIV and their DICGs to produce income [16,23].

Social factors relating to poverty often increase the risk of HIV transmission and interfere with a person's ability to cope with the disease. The social position of women in many societies is linked to their increased susceptibility to HIV/AIDS. In resource-poor settings worldwide, women tend to suffer a greater burden from poverty since, as demonstrated in the current study, they do not have as much social freedom as men [13,30,33,34]. Women are more frequently driven to prostitution or early marriage, are more susceptible to sexual violence and trafficking [38], and tend to have less control over decisions related to sexuality and disease prevention, such as refusing sex or using protection [34].

In addition to being more susceptible to HIV/AIDS, women predominately assume the role of caregiver due to prevailing patriarchal attitudes and social structure. The stress of care giving is thus much greater on women than on men. Once the PLHIV has passed away, the female caregiver (who may herself be HIV positive) is then left with few means of support, having exhausted all resources in the care of the PLHIV.

Finally, due to the low status of women, access to care and support for female PLHIV can be particularly difficult. The unique issues of women and HIV/AIDS must be addressed through a formal CHBC programme. It is important to develop strategies to reduce the complex and multifaceted burden of HIV/AIDS on women [13,17,23-27].

Informal care must be supplemented with formal care in order to decrease the strain on the informal caregiver [16,23-27]. Consistent with the current literature [9,10,18], female family members were found to be primary caregivers of PLHIV. All PLHIV reported receiving little support from friends and community members. The men, in particular, reported that they had "no friends" and no support outside of their family. DICGs also discussed isolation and lack of support as they struggled to care for the PLHIV. The drain on family caregivers is often extensive and can negatively impact their mental and physical health [21].

This descriptive study provides the foundation for the development of a model of CBHC that is sensitive to the expressed needs of PLHIV and their DICGs in the rural Eastern Cape, South Africa. To meet the needs identified by PLHIV in this study, the basic services offered by a home-based care programme in the Eastern Cape must include: in-home assistance to monitor health status; physical, emotional, and financial support; basic medical care and symptom control; medication, food and supplies delivery; and counselling and education regarding proper care. CBHC programmes must also provide training to volunteers and caregivers in basic health care and emotional counselling to support DICGs and alleviate their burden of care.

## Study limitations

In interpreting these findings, study limitations should be noted. The nine PLHIV who participated in this study were primarily unmarried women. None of the participants were using antiretroviral therapy. Caution should be used, therefore, in applying the results of this study to male PLHIV, or to PLHIV who are using antiretroviral therapy.

It is also important to note that, due to the established criteria for selection of the interviewees, only PLHIV who had disclosed their status to at least one family member who was willing to care for them were eligible for participation. Therefore, the current findings do not reflect the issues facing those PLHIV who have not disclosed their status to their families, or whose families have rejected them as a result of their status.

The members of the research team relied exclusively on the interpreters to identify the PLHIV and their caregivers. The small number of participants and the use of convenience sampling reflected the small numbers of PLHIV who have disclosed their status. The small number of male participants reflected the fact that few numbers of men in the community have disclosed their HIV-positive status. Additionally, a number of men who were approached stated that they were not comfortable participating in the project.

## Conclusions

In-depth data regarding the needs of PLHIV and their DICGs living in a rural African community were collected. These needs were explored with the purpose of informing the development of strategies that will enable those affected to cope with the disease. Data from this study, combined with relevant literature, has identified the needs of PLHIV and their DICGs. This information can serve as the basis for the development of a CBHC programme.

The community needs assistance to build its capacity to provide comprehensive support for PLHIV and their caregivers. Assistance must come from a variety of sectors, including government, non-governmental organizations and businesses. The development of supplementary health care programmes and increased levels of support for PLHIV and their caregivers will empower thousands of people to cope with HIV/AIDS and its complex psychological and social challenges.

## Competing interests

The authors declare that they have no competing interests.

## Authors' contributions

BM and NM contributed equally to the conception, design and implementation of the study. Both authors read and approved this manuscript.
